# Retrospective Epidemiological Analyses of 12,976 Culture‐Positive Superficial Fungal Infections in Shanghai, East China

**DOI:** 10.1111/myc.70164

**Published:** 2026-03-04

**Authors:** Chunjiao Zheng, Wenjing He, Jingwen Tan, Xiaoping Liu, Yuanyuan Wang, Lulu Li, Linan Ni, Qian Yu, Lianjuan Yang

**Affiliations:** ^1^ Department of Medical Mycology, Center of Infectious Skin Diseases, Shanghai Skin Disease Hospital Tongji University School of Medicine Shanghai China

**Keywords:** *Candida*, dermatophytes, *Malassezia*, superficial fungal infections, tinea

## Abstract

**Background:**

Superficial fungal infections (SFIs) are common dermatological conditions, and both domestic and international reports indicate an increasing incidence in recent years. Their prevalence is strongly affected by climate, temperature, humidity and lifestyle, resulting in significant regional variations.

**Objective:**

To describe the epidemiological characteristics of superficial fungal infections in Shanghai, East China, from 2022 to 2024.

**Methods:**

This single‐centre retrospective observational study was conducted at Shanghai Skin Disease Hospital. Clinical specimens, including skin scales, hair and nails, were examined using KOH microscopy, followed by culture on Sabouraud dextrose agar. Fungal identification was primarily based on colony morphology and microscopic features.

**Results:**

A total of 12,976 cases of superficial fungal infections were recorded during the study period. Onychomycosis was the most common infection (33.75%), followed by tinea cruris (20.05%) and tinea pedis (18.63%). Significant gender differences were identified: females showed higher rates of onychomycosis and tinea pedis, while males more frequently presented with tinea cruris, tinea corporis and other SFIs. The highest number of infections occurred in individuals aged 31–40 years, with onychomycosis, tinea pedis and tinea versicolor being most prevalent in this group. Among culture‐positive samples, dermatophytes accounted for the majority (52.54%), with *Trichophyton rubrum* being the most frequently isolated species (44.27%). Dermatophyte infections were more common in males, whereas yeast infections were more frequent in females. Dermatophyte infections peaked in the 31–40 and 61–70 age groups, while yeast infections were most common among individuals aged 21–40.

**Conclusion:**

This study provides valuable epidemiological insights into superficial fungal infections in Shanghai, offering necessary guidance for clinical diagnosis, treatment decision‐making and prevention strategies.

## 1 Introduction

Superficial fungal infections (SFIs) represent a common dermatological issue, with recent studies reporting a continuous increase in their incidence [1, 2, 3, 4]. Their occurrence is strongly influenced by climatic factors, particularly temperature, humidity and lifestyle habits, resulting in significant regional differences [2, 5, 6, 7]. The primary causative agents include dermatophytes, yeasts and moulds.

In China, most existing epidemiological research has focused on specific fungal diseases, and comprehensive data on the overall epidemiology of dermatophytosis remain limited. Shanghai was selected as the study site for three key reasons that highlight its unique epidemiological relevance. First, the city has a subtropical monsoon climate marked by high humidity, hot, rainy summers and clear seasonal fluctuations. Second, the Shanghai Skin Disease Hospital, the largest specialised dermatological institution in East China, serves as a major referral centre with more than one million outpatient visits annually. Its patient population is diverse, drawn not only from Shanghai but also from surrounding provinces such as Jiangsu, Zhejiang and Anhui. Third, despite rapid urbanisation and lifestyle changes that may influence SFIs' distribution, recent large‐scale epidemiological data for Shanghai remain insufficient.

To address this gap, this study collected skin, hair and nail specimens from patients diagnosed with superficial fungal infections between January 2022 and September 2024. These samples were subjected to microscopic examination and fungal culture, followed by statistical analysis. This represents the first study to systematically assess the spectrum and clinical types of superficial fungal infections in Shanghai, East China.

## 2 Materials and Methods

### 2.1 Study Area Characteristics

This study was conducted in Shanghai, a major metropolitan city in eastern China. The region experiences a subtropical monsoon climate characterised by high humidity, pronounced seasonal variations and frequent rainfall. Summer temperatures typically range from 28°C to 38°C (http://data.cma.cn/), creating a warm and moist environment favourable for fungal growth. Lifestyle factors such as high population density, increased indoor activity associated with urbanisation, and frequent use of shared public facilities (e.g., swimming pools and gyms) may further contribute to the spread of superficial fungal infections. Socioeconomic factors, including access to healthcare, educational levels and certain occupations (e.g., manual labourers at higher risk of dermatophyte exposure) may also influence infection patterns. These regional characteristics were taken into account when interpreting the epidemiological findings of superficial fungal infections.

### 2.2 Clinical Data

Data were collected from patients diagnosed with superficial fungal infections who visited the Shanghai Skin Disease Hospital between January 2022 and September 2024, except for February, April and May 2022 due to COVID‐19 lockdowns. Patients with clinically suspected SFIs who underwent both microscopic examination and fungal culture were included. Cases with contaminated cultures or incomplete clinical information were excluded. This study was observational in design, and the protocol was approved by the Research Ethics Committee of the Shanghai Skin Disease Hospital (Approval No. 2025–34).

### 2.3 Sample Collection and Identification Methods

Lesion samples were collected using sterilised blunt knives to scrape scales from the margins of affected areas. Nail material was obtained by scraping with a sterile scalpel. For hair infections, approximately 10 infected hairs were collected, and the surrounding follicular scales were scraped or compressed using tweezers. Samples were placed on glass slides, treated with a fluorescent stain, coverslipped and examined microscopically. Portions of each specimen were subsequently inoculated onto Sabouraud dextrose agar (SDA) containing chloramphenicol and incubated at 26°C for four weeks, with observations performed twice weekly.

Fungal identification was based on macroscopic colony characteristics, including morphology, pigmentation and growth appearance, as well as microscopic features. For detailed examination of mould structures, a sellotape touch preparation stained with lactophenol cotton blue was used. In cases of suspected pityriasis versicolor or folliculitis, skin scrapings showing the characteristic ‘spaghetti and meatballs’ pattern under 10% KOH were cultured on SDA slants overlaid with sterile olive oil and incubated at 30°C. These cultures were monitored daily for *Malassezia* growth. CHROMagar medium was also used when necessary to differentiate certain *Candida* species.

Dermatophyte differentiation, particularly among *Trichophyton* species, relied on macroconidia morphology and colony pigmentation. For non‐dermatophyte moulds, only organisms generally associated with superficial mycoses, especially nail infections, were identified, and their clinical significance was assessed using three criteria: (i) direct microscopy showing irregular, broad, tortuous septate hyphae; (ii) positive culture defined as pure growth of the same mould at all inoculation points and (iii) when possible, consistent isolation of the same organism from two separate specimens collected 2–3 weeks apart [8]. The identification process incorporated direct microscopic findings, colony morphology and the clinical relevance of isolated fungi. Furthermore, lesion type and patient symptoms were correlated with laboratory results to improve clinical interpretation.

### 2.4 Statistical Methods

Data analysis and visualisation were performed using SPSS 26.0. Categorical variables were presented as counts (*n*) and percentages (%), and gender differences were assessed using chi‐square tests. A *p*‐value of < 0.05 was considered statistically significant.

## 3 Results

### 3.1 Distribution of Infection Types and Patient Demographics

During the study period, 32,390 patients were clinically suspected of having superficial fungal infections. Of these, 12,976 cases (40.06%) with positive microscopy and culture results were included in the analysis. The cohort comprised 7130 males and 5846 females, yielding a male‐to‐female ratio of 1.22:1. Patient ages ranged from under 1 year (recorded as 0) to 98 years, with a mean age of 45.37 ± 18.44 years. Nine types of superficial fungal infections were identified. Onychomycosis was the most prevalent (4380 cases, 33.75%), followed by tinea cruris (2602 cases, 20.05%), tinea pedis (2418 cases, 18.63%), tinea corporis (1261 cases, 9.72%), tinea manuum (813 cases, 6.27%), folliculitis (658 cases, 5.07%), tinea versicolor (520 cases, 4.01%), tinea faciei (274 cases, 2.11%) and tinea capitis (50 cases, 0.39%).

### 3.2 Comparison of Infection Types by Gender

Among females, onychomycosis was the most common infection, followed by tinea pedis and tinea cruris. In comparison, males most frequently presented with tinea cruris, followed by onychomycosis and tinea pedis. Onychomycosis and tinea pedis were more prevalent in females, whereas males showed higher rates of tinea cruris, tinea corporis, tinea manuum, folliculitis and tinea versicolor. These gender‐related differences were statistically significant (*p* < 0.05). No significant gender differences were found for tinea faciei or tinea capitis (Table 1).

**TABLE 1:** Comparison of different infectious diseases by gender.

Disease	Female *n* (%)	Male *n* (%)	*χ* ^2^	*p*
Onychomycosis	2582 (19.90)	1798 (13.86)	515.842	< 0.001
Tinea cruris	553 (4.26)	2049 (15.79)	744.683	< 0.001
Tinea pedis	1310 (10.10)	1108 (8.54)	99.949	< 0.001
Tinea corporis	516 (3.98)	745 (5.74)	9.635	0.002
Tinea manuum	309 (2.38)	504 (3.88)	17.390	< 0.001
Tinea faciei	112 (0.86)	162 (1.25)	1.972	0.160
Tinea capitis	29 (0.22)	21 (0.16)	3.399	0.065
Folliculitis	242 (1.86)	416 (3.21)	19.170	< 0.001
Tinea Versicolor	193 (1.49)	327 (2.52)	13.785	< 0.001
Total	7130 (54.95)	5846 (45.05)	—	—

### 3.3 Fungal Infection Rates in Various Age Groups

**FIGURE 1:**
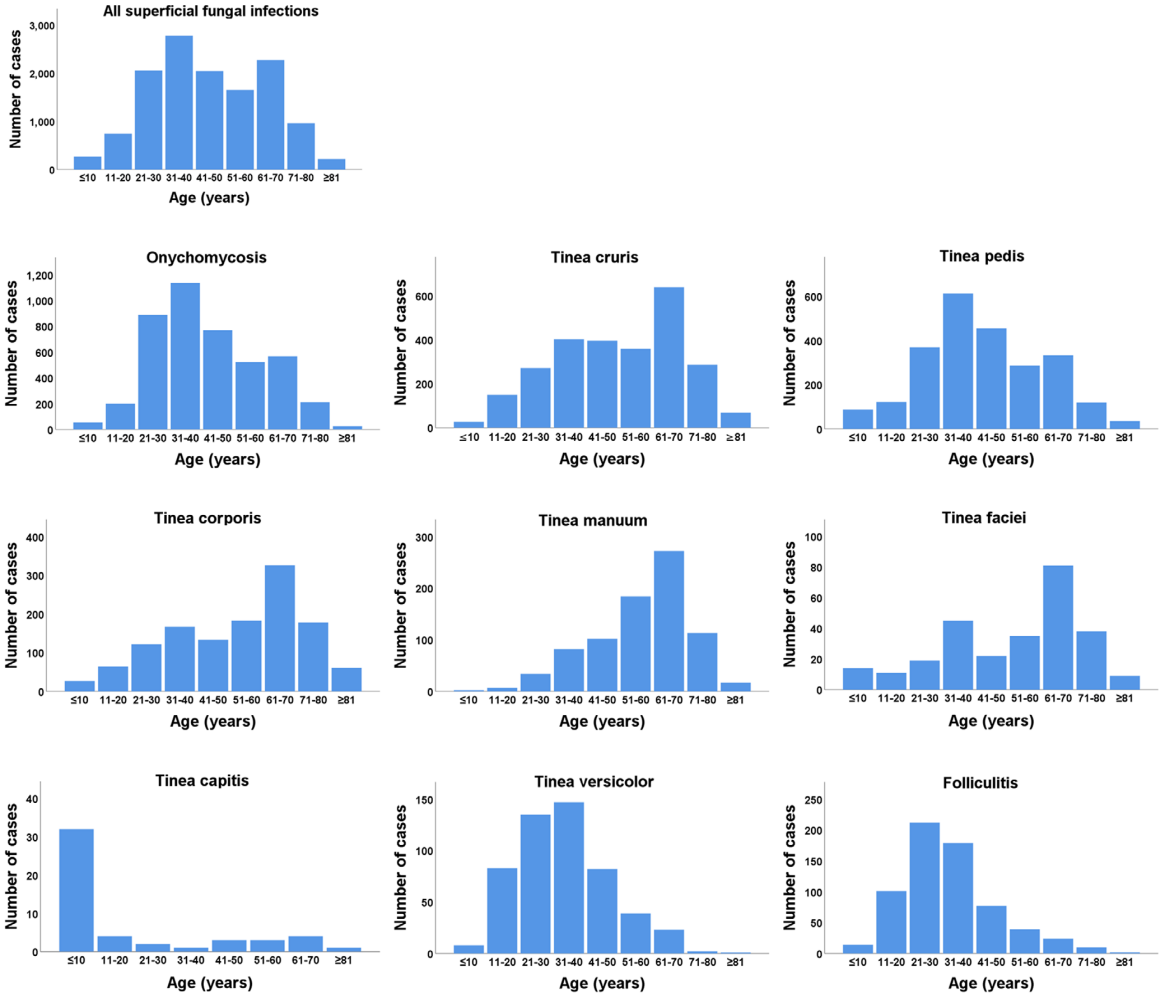
Superficial fungal infection distributions by patient age.

As illustrated in Figure 1, the highest number of infections occurred in the 31–40 age group (2773 cases), followed by the 61–70 age group (2271 cases). Infections were comparatively uncommon among individuals aged 10 years or younger and those aged 80 years or older. Onychomycosis, tinea pedis and tinea versicolor were most frequently observed in the 31–40 age group, whereas tinea cruris, tinea corporis, tinea manuum and tinea faciei were more common among individuals aged 61–70. Folliculitis was most prevalent in the 21–30 age group, while tinea capitis occurred most often in the youngest age group.

### 3.4 Pathogenic Fungal Distributions and Gender Differences

Among the 12,976 culture‐positive specimens, dermatophytes were the most frequently identified pathogens (6818 strains, 52.54%), followed by yeasts (3239 strains, 24.96%) and moulds (2919 strains, 22.50%). Fourteen pathogenic species were detected, including three classified as ‘others’ due to unidentified dermatophytes, yeasts or moulds. *Trichophyton rubrum* ( *T. rubrum* ) was the predominant species (5744 strains, 44.27%), while *Trichophyton tonsurans* (*T. tonsurans*) was the least common (4 strains, 0.03%). Among the yeasts, *Candida* was the most prevalent genus (1422 cases, 10.96%), with *Candida albicans* as the leading species. Only a minimal number of *Candida glabrata* and *Candida tropicalis* were detected, and no *Candida krusei* was identified during the study period. *Aspergillus* was the most common mould (1187 strains, 9.15%) (Table 2).

**TABLE 2:** Species composition of pathogenic fungi and gender‐based comparative analysis.

Pathogenic fungi	Count	Percentage (%)	Female *n* (%)	Male *n* (%)	*χ* ^2^	*p*
Dermatophytes			2848 (21.95)	3970 (30.59)	62.460	< 0.001
*T. rubrum*	5744	44.27	2257 (17.39)	3487 (26.87)	138.089	< 0.001
Other *Trichophyton* species	615	4.74	301 (2.32)	314 (2.42)	3.948	0.047
*T. mentagrophytes*	197	1.52	119 (0.92)	78 (0.60)	19.049	< 0.001
*M. canis*	190	1.46	145 (1.12)	45 (0.35)	76.132	< 0.001
*E. floccosum*	56	0.43	18 (0.14)	38 (0.29)	3.786	0.052
*N. gypsea*	6	0.05	4 (0.03)	2 (0.02)	0.419	0.256
*T. violaceum*	6	0.05	3 (0.02)	3 (0.02)	1.000	0.560
*T. tonsurans*	4	0.03	1 (0.01)	3 (0.02)	0.632	0.390
Yeasts			1466 (11.30)	1773 (13.66)	0.076	0.783
*Candida*	1422	10.96	684 (5.27)	738 (5.69)	5.994	0.014
*Malassezia*	1178	9.08	435 (3.35)	743 (5.73)	34.194	< 0.001
Other Yeasts	639	4.92	347 (2.67)	292 (2.25)	23.236	< 0.001
Moulds			1532 (11.81)	1387 (10.69)	84.018	< 0.001
*Aspergillus*	1187	9.15	634 (4.89)	553 (4.26)	36.882	< 0.001
*Penicillium*	834	6.43	437 (3.37)	397 (3.06)	19.427	< 0.001
Other filamentous moulds	898	6.92	461 (3.55)	437 (3.37)	15.389	< 0.001
Total	12,976	100	5846	7130	—	—

Gender‐based differences were evident in the distribution of pathogenic fungi. Males had higher rates of dermatophyte infections, whereas females showed higher rates of yeast infections. Males were more frequently infected with *T. rubrum* , *Candida* and *Malassezia* (*p* < 0.05), while females had a higher prevalence of *Trichophyton mentagrophytes* (*T. mentagrophytes*), *Microsporum canis* ( *M. canis* ) and moulds (*p* < 0.05). Infection rates of *Epidermophyton floccosum* (*E. floccosum*), *Nannizzia gypsea* (*N. gypsea*), *Trichophyton violaceum* ( *T. violaceum* ) and *T. tonsurans* did not differ significantly between genders (Table 2).

### 3.5 Associations Between Pathogenic Fungi and Patient Age

**FIGURE 2:**
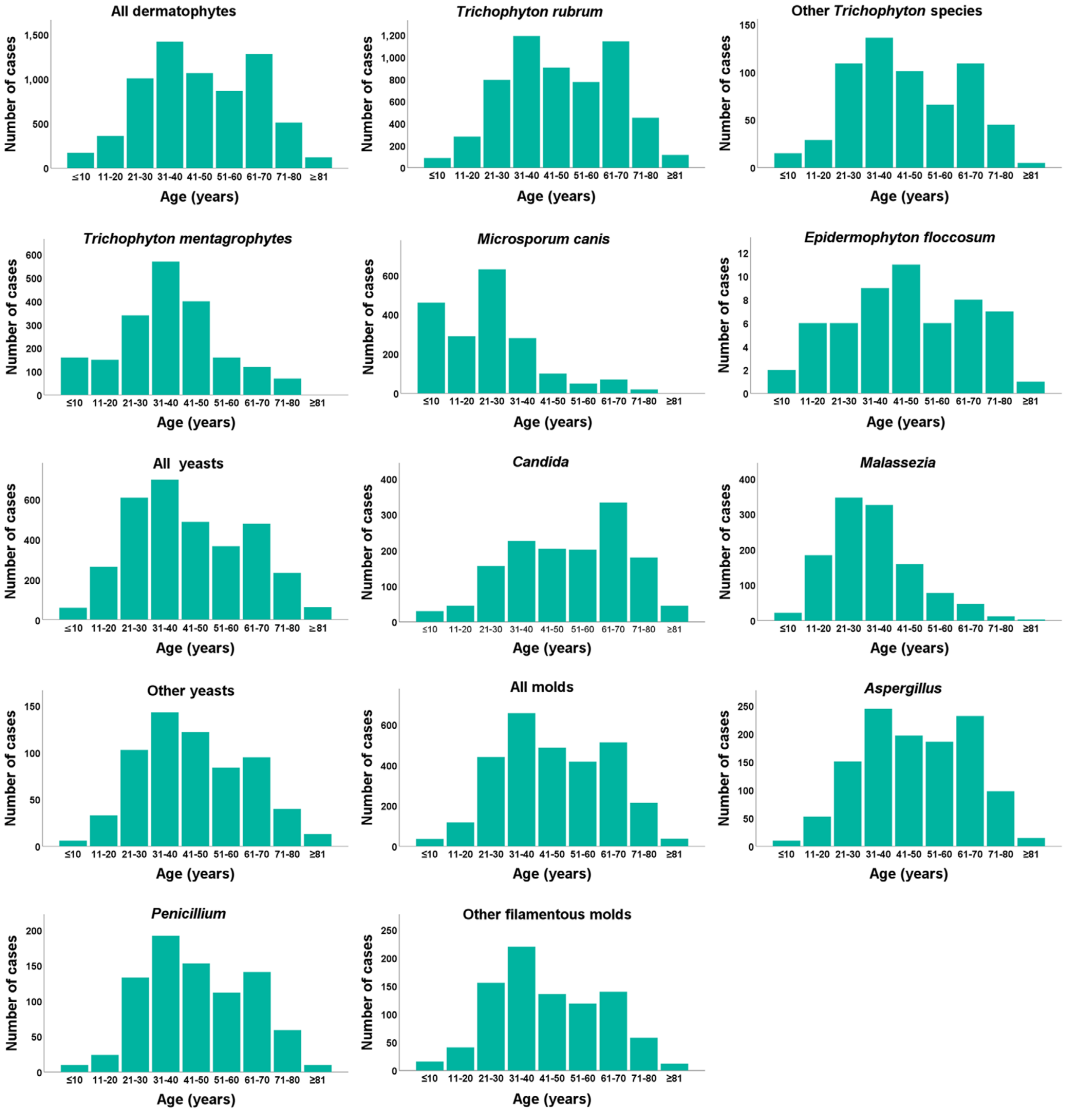
Distribution of pathogenic species based on patient age. No graphs were prepared for *N. gypsea*, *T. violaceum* or *T. tonsurans* due to the relatively limited case numbers.

Dermatophyte infections were most common in individuals aged 31–40 and 61–70 years. *T. rubrum* , other dermatophytes and *T. mentagrophytes* were predominantly detected in the 31–40 age group, whereas *M. canis* occurred mainly in patients aged 30 years or younger. *E. floccosum* showed its highest prevalence among those aged 41–50 years. Yeast infections, including *Malassezia* spp., were most frequently observed in individuals aged 21–40, whereas *Candida* infections were more common in individuals aged 61–70. Mould infections were similarly concentrated in the 31–40 age range (Figure 2).

### 3.6 Associations Between Infection Types and Pathogenic Fungi

As shown in Table 3, the most frequently detected pathogenic fungi were *T. rubrum* , *Candida* spp. and *Aspergillus* spp., which were most closely associated with onychomycosis, tinea cruris and tinea pedis, respectively. *M. canis* was predominantly linked to tinea corporis, tinea manuum and tinea capitis. *E. floccosum* was mainly associated with tinea cruris and tinea pedis, while *N. gypsea* and *T. violaceum* were identified in cases of tinea corporis and tinea capitis. *T. tonsurans* was occasionally observed in tinea faciei, tinea corporis and tinea capitis. *T. rubrum* was the principal pathogen responsible for onychomycosis, tinea cruris, tinea pedis, tinea corporis, tinea manuum and tinea faciei, whereas *M. canis* was the leading cause of tinea capitis. Tinea versicolor and folliculitis were primarily attributed to *Malassezia* spp.

**TABLE 3:** Relationship between various diseases and pathogenic fungi.

Pathogenic fungi	Onychomycosis	Tinea cruris	Tinea pedis	Tinea corporis	Tinea manuum	Tinea faciei	Tinea capitis	Folliculitis	Tinea versicolor	Total
Dermatophytes										
*T. rubrum*	1562	1769	1142	744	378	143	6	0	0	5744
Other *Trichophyton* species	227	130	124	78	28	25	3	0	0	615
*T. mentagrophytes*	34	11	121	13	5	10	3	0	0	197
*M. canis*	1	8	1	127	3	21	29	0	0	190
*E. floccosum*	1	18	31	2	4	0	0	0	0	56
*N. gypsea*	0	0	0	4	0	0	2	0	0	6
*T. violaceum*	0	2	0	1	0	0	3	0	0	6
*T. tonsurans*	0	0	0	1	0	2	1	0	0	4
Yeasts										
*Candida*	543	377	192	117	172	20	1	0	0	1422
*Malassezia*	0	0	0	0	0		0	658	520	1178
Other Yeasts	382	54	130	32	41	0	0	0	0	639
Moulds										
*Aspergillus*	720	74	254	50	69	19	1	0	0	1187
*Penicillium*	443	71	209	34	61	15	1	0	0	834
Other filamentous moulds	467	88	214	58	52	19	0	0	0	898
Total	4380	2602	2418	1261	813	274	50	658	520	12,976

## 4 Discussion

The Shanghai Skin Disease Hospital is situated in a rapidly developing coastal region of eastern China, characterised by high population density, a substantial migrant worker presence and convenient transportation networks. As a result, the hospital receives patients from both Shanghai and neighbouring provinces. In this study, analysis of individuals clinically suspected of superficial fungal infections revealed a male‐to‐female ratio of 1.22:1. These results are in good agreement with previously reported studies [7, 8, 9, 10, 11, 12]. The most frequently diagnosed infections were onychomycosis, tinea cruris and tinea pedis, a pattern comparable to that reported in Hangzhou, Southeast China [11]. However, significant differences emerged when comparing data from other regions. Studies from Guangzhou, southern China, reported tinea cruris, tinea capitis and tinea pedis as the predominant infections [7]. Those from Kinshasa in the Democratic Republic of Congo identified tinea corporis, tinea capitis and tinea pedis as the most common [13]. In Cayenne, French Guiana, onychomycosis, tinea capitis and tinea pedis were the leading presentations [14]. In comparison, in Japan, tinea pedis, onychomycosis and tinea corporis were most prevalent. These variations highlight the influence of regional environmental, socioeconomic and demographic factors on the distribution of superficial fungal infections [15].

Gender‐specific differences in the incidence of superficial fungal infections were clearly observed. Tinea cruris, tinea corpori*s*, tinea manuum, folliculitis and tinea versicolor were more frequently diagnosed in males, while onychomycosis was more common in females. These findings are consistent with previous domestic and international reports [7, 11, 16, 17]. The higher prevalence of certain fungal infections in males may be attributed to lifestyle and physiological factors [18, 19]. Elevated androgen levels can increase sebum secretion and perspiration, creating a slightly alkaline skin environment favourable for fungal growth. Similarly, men are more likely to engage in physical labour and outdoor activities, which heighten exposure to environmental fungi, and they may pay less attention to personal hygiene [20, 21, 22]. The greater incidence of onychomycosis and tinea pedis among females may relate to nail barrier damage from household chores, frequent nail treatments involving chemical agents and the use of poorly ventilated footwear, such as high heels and boots, that promotes sweating and moisture retention. Moreover, women may be more inclined to seek medical care due to heightened concern about cosmetic appearance [1, 11, 22, 23].

Age‐related differences in infection patterns were also evident. The highest incidences of onychomycosis, tinea pedis and tinea versicolor were observed in individuals aged 31–40 years, while folliculitis was most common in the 21–30 age group. Adults aged 21–40 represent the primary working population, characterised by active social interactions, increased perspiration and frequent exposure to environmental fungi. This age group also tends to demonstrate strong physiological activity, including elevated hormone and sebum production, which creates a skin microenvironment conducive to fungal growth. Shanghai's subtropical monsoon climate further promotes fungal proliferation, and the city's economic development and extensive population mobility may contribute to the higher susceptibility of younger individuals to various infections, including those caused by *Malassezia* spp. In comparison, individuals aged 61–70 years showed increased rates of tinea cruris, tinea corporis, tinea manuum and tinea faciei.

Tinea capitis is most commonly seen in children, likely due to their underdeveloped sebaceous glands and relatively immature immune systems [24, 25, 26]. *M. canis* was identified as the predominant pathogen causing tinea capitis, consistent with findings from both domestic and international studies [25, 26, 27, 28, 29, 30]. This zoophilic species is frequently carried by household pets such as cats and dogs and can readily infect children who have close contact with these animals [24, 26].

In this study, 14 pathogenic species were isolated, with *T. rubrum* being the most prevalent, an observation that aligns with previous research [6, 10, 31]. A significant number of *Candida* infections was also detected, which may be attributed to an ageing population and the widespread use of broad‐spectrum antibiotics, corticosteroids and immunosuppressive therapies [32, 33]. These trends underscore the growing importance of recognising and managing *Candida* infections.

Moulds, widely recognised as important global pathogens, were primarily associated with onychomycosis in this study. The prevalence of mould‐related infections varies considerably across regions [3]. Studies from Brazil [34], Sri Lanka [35] and Thailand [36] have reported mould involvement in approximately 60% of cases, whereas rates in Europe are substantially lower, at around 5% [3]. In tropical climates such as Thailand, *Trichophyton* spp. are the predominant pathogens [36, 37], while in Europe, *Scopulariopsis brevicaulis, Aspergillus* spp., *Acremonium* spp. and *Fusarium* spp. are more common [3]. In this study, *Aspergillus* spp. were the most commonly identified moulds, predominantly causing onychomycosis. These pronounced regional variations likely reflect differences in climate and environmental conditions, as well as socioeconomic and lifestyle factors [38].

This study has several limitations. First, as a single‐centre investigation, the generalisability of the findings may be restricted. Second, auxiliary diagnostic methods incorporating molecular identification were not included. Hopefully, our ongoing research integrates advanced technologies, including a novel artificial intelligence (AI)‐based fluorescence microscopic image analyser (FMIA) [39], MALDI‐TOF MS and combines them with PCR, to develop a comprehensive fungal strain library. This effort will not only reinforce the foundation for future studies but also significantly improve diagnostic accuracy in clinical mycology.

It is also important to recognise that the epidemiology of superficial fungal infections is dynamic and evolves alongside demographic and medical shifts. As China transitions into an ageing society, the proportion of older adults, who are more vulnerable to onychomycosis and *Candida* infections due to comorbidities and age‐related immune decline is expected to rise [21, 32]. At the same time, the increasing use of immunosuppressive therapies and broad‐spectrum antibiotics may further influence host‐pathogen interactions and alter the spectrum of superficial fungal diseases [40, 41, 42]. As a result, our findings reflect a cross‐sectional snapshot of current epidemiological patterns. A decade from now, pathogen distribution, affected age groups and clinical presentations may differ significantly, just as our results already diverge from reports published in previous decades [21]. These observations underscore the importance of continuous long‐term surveillance to track evolving trends and anticipate emerging challenges in the prevention and management of superficial fungal infections.

## Author Contributions

Lianjuan Yang and Qian Yu designed this study. Chunjiao Zheng and Wenjing He performed the data analysis and visualisation. Chunjiao Zheng wrote the paper. Jingwen Tan, Xiaoping Liu, Yuanyuan Wang, Lulu Li and Linan Ni contributed to the analysis and interpretation of data. All authors read and approved the final manuscript. Lianjuan Yang and Qian Yu were primarily responsible for the final content.

## Conflicts of Interest

The authors declare no conflicts of interest.

## Data Availability

The data that support the findings of this study are available from the corresponding author upon reasonable request.
