# Correction to ‘Retrospective Epidemiological Analyses of 12,976 Culture‐Positive Superficial Fungal Infections in Shanghai, East China’

**DOI:** 10.1111/myc.70215

**Published:** 2026-08-04

C. Zheng , W. He , J. Tan , et al., “Retrospective Epidemiological Analyses of 12,976 Culture‐Positive Superficial Fungal Infections in Shanghai, East China,” Mycoses 69, no. 3 (2026): e70164, 10.1111/myc.70164.41777220 PMC12958013

In the Abstract and Section 3.4, the description of gender differences in pathogenic fungal distribution was inaccurate. In the Abstract, the text ‘Dermatophyte infections were more common in males, whereas yeast infections were more frequent in females’ was incorrect. It should read: ‘Dermatophyte infections were more common in males, whereas mould infections were more frequent in females’. In Section 3.4, the text ‘Males had higher rates of dermatophyte infections, whereas females showed higher rates of yeast infections’ was incorrect. It should read: ‘Males had higher rates of dermatophyte infections, whereas females showed higher rates of mould infections’.

We apologize for this error.

